# Molecular types, clinical profile, and exploratory virulence-associated phenotypes of clinical *Cryptococcus* isolates from Amazonas, Brazil

**DOI:** 10.1007/s42770-026-02094-5

**Published:** 2026-09-30

**Authors:** Robert Langlady Lira Rosas Filho, Izabela de Mesquita Bárcia Moreira, Nayara de Fátima Lazameth-Diniz, Flávia Fernandes Souza, João Flávio Dias do Val Bisneto, Naira Sulany Oliveira de Sousa, Juan Diego Ribeiro de Almeida, Érica Simplício de Souza, Kátia Santana Cruz, Carla Silvana da Silva Santos, Hagen Frickmann, João Vicente Braga de Souza

**Affiliations:** 1 Graduate Program in Pharmaceutical Sciences, Federal University of Amazonas, Manaus, Amazonas Brazil; 2 Laboratory of Mycology, National Institute for Amazonian Research, Manaus, Amazonas Brazil; 3 State University of Amazonas, Manaus, Amazonas Brazil; 4 Dourado Tropical Medicine Foundation, Manaus, Amazonas Brazil; 5 Department of Microbiology and Hospital Hygiene, Bundeswehr Hospital Hamburg, Hamburg, Germany; 6 Institute for Medical Microbiology, Virology and Hygiene, University Medicine Rostock, Rostock, Germany

**Keywords:** *Cryptococcus* spp, Cryptococcosis, Molecular typing, URA5 PCR-RFLP, Brazilian Amazon, Virulence-associated phenotypes

## Abstract

**Background:**

Cryptococcosis remains a severe systemic mycosis, especially among people living with HIV/AIDS, and culture-based molecular data from Amazonas remain limited.

**Objectives:**

The aim was to identify which *Cryptococcus* molecular types circulated among clinical isolates from Amazonas between 2020 and 2024; what were the main clinical characteristics and outcomes of patients infected with *Cryptococcus neoformans* molecular type VNI and *Cryptococcus gattii* molecular type VGII; and whether these molecular types differed in selected susceptibility-related and virulence-associated phenotypes.

**Methods:**

We analyzed 70 culture-confirmed clinical isolates from patients treated at the Dr. Heitor Vieira Dourado Tropical Medicine Foundation, Manaus, Brazil. Molecular types were assigned by URA5 PCR-RFLP using reference strains. Demographic and clinical variables were retrieved from medical records. A stratified 30-isolate subset comprising 24 VNI and 6 VGII isolates was evaluated for phospholipase activity and melanization. Fluconazole MICs were analyzed for the complete 70-isolate dataset and were determined by broth microdilution according to CLSI M27-A4.

**Results:**

VNI predominated (64/70; 91.4%), whereas VGII accounted for 6/70 isolates (8.6%). The series was predominantly composed of men, young adults, and HIV-positive patients. HIV infection was more frequent among VNI cases, whereas VGII included proportionally more HIV-negative patients. In-hospital death occurred in 17/70 patients (24.3%). Fluconazole MICs, phospholipase activity, and melanization were broadly comparable between VNI and VGII, although the small VGII subgroup precluded robust genotype-associated inference.

**Conclusion:**

VNI was the dominant molecular type in this severe reference-center cryptococcosis series from Amazonas. Phenotypic findings should be interpreted as exploratory and hypothesis-generating.

**Supplementary Information:**

The online version contains supplementary material available at https://doi.org/10.1007/s42770-026-02094-5.

## Introduction

Cryptococcosis is a life-threatening systemic mycosis and remains one of the most important invasive fungal infections among people living with HIV/AIDS and other immunocompromised populations [1–3]. Infection usually begins after inhalation of environmental propagules belonging to the *Cryptococcus neoformans*/*Cryptococcus gattii* species complexes [4, 5]. The pulmonary phase may remain asymptomatic or progress to disseminated disease, with marked tropism for the central nervous system. In Amazonas, where severe cryptococcosis is treated in regional reference services, viable isolates linked to clinical data are essential for local surveillance.

Molecular studies have demonstrated substantial genetic diversity among clinical and environmental isolates of *Cryptococcus* spp. PCR-RFLP, AFLP, multilocus sequence typing, and whole-genome sequencing have enabled the recognition of major molecular types and their association with geography, host status, antifungal susceptibility, and clinical presentation [6–11]. Although MLST and whole-genome sequencing provide higher discriminatory power, URA5 PCR-RFLP remains useful for regional surveillance when reference strains are included and genomic infrastructure is limited [9]. In parallel, cryptococcal pathogenicity has been linked to capsule production, melanization, extracellular enzymes, growth at physiological temperature, and host adaptation mechanisms [12–19].

Despite advances in the characterization of *Cryptococcus* spp. in several Brazilian regions, culture-based molecular data from the Brazilian Amazon remain limited. Previous Latin American and Brazilian studies have shown that *C. neoformans* VNI predominates among clinical isolates, whereas VGII remains epidemiologically relevant in clinical and environmental contexts [7–11]. However, few studies have integrated molecular typing, clinical information, fluconazole minimum inhibitory concentration (MIC) records, and exploratory virulence-associated phenotypes of clinical isolates from Amazonas.

Thus, the study was organized around three practical questions: (i) which molecular types were present among clinical *Cryptococcus* spp. isolates from Amazonas between 2020 and 2024; (ii) what demographic and clinical profile, including HIV status, CD4 count, ICU admission, recurrence, and hospital outcome, characterized patients infected by VNI and VGII; and (iii) whether these molecular types differed in selected fluconazole susceptibility-related and virulence-associated phenotypes. The primary objective was to determine the molecular types of 70 clinical isolates. The secondary objectives were to describe the patient profile according to molecular type and to explore fluconazole minimum inhibitory concentration (MIC) records, phospholipase activity, and melanization.

## Materials and methods

The methods were organized according to the three questions of the study: molecular typing of the isolates, clinical characterization of the corresponding patients, and exploratory comparison of selected phenotypic traits between the main molecular types.

### Study design and study site

This was a retrospective observational study of clinical *Cryptococcus* spp. isolates and associated medical-record data from patients treated at the Dr. Heitor Vieira Dourado Tropical Medicine Foundation (FMT-HVD), Manaus, Amazonas, Brazil. Molecular and phenotypic procedures were performed at the Laboratory of Mycology, National Institute for Amazonian Research (INPA). A case was defined as a patient with cryptococcosis and recovery of a viable *Cryptococcus* spp. isolate from routine clinical laboratory diagnosis. The study was approved by the Research Ethics Committee under CAAE No. 90749718.3.0000.0005.

### Clinical isolates and inclusion criteria

The study evaluated 70 clinical isolates recovered from cerebrospinal fluid (CSF) of patients with neurocryptococcosis between 2020 and 2024 and previously identified as *Cryptococcus* spp. by the FMT-HVD laboratory using routine phenotypic methods. The final dataset included one viable clinical *Cryptococcus* isolate per patient episode. Because the specimen source was uniform, it was not included as a variable in genotype-associated comparisons. Inclusion criteria were isolate viability, year of isolation and confirmation as *Cryptococcus* spp. Duplicate isolates from the same episode, non-viable isolates, and isolates with inconclusive molecular profiles after repeated testing were excluded from the respective analyses.

### Reference strains used for molecular typing

Reference strains representing the major molecular types were obtained from Fundação Oswaldo Cruz, Brazil: *C. neoformans* WM 148 (VNI), WM 626 (VNII), WM 628 (VNIII), WM 629 (VNIV), *C. gattii* WM 179 (VGI), WM 178 (VGII), WM 175 (VGIII), and WM 779 (VGIV). These strains were used as molecular-type controls for URA5 PCR-RFLP profile comparison and supported the specific objective of assigning molecular types to clinical isolates.

### Clinical characterization according to molecular type

Clinical and epidemiological data were retrospectively collected from medical records of patients diagnosed with cryptococcosis and treated at FMT-HVD. Variables included sex, age group, HIV infection status, initial CD4 + T-lymphocyte count when available, ICU admission, hospital outcome, and recurrence. Clinical variables were analyzed only for patients with available medical-record data, and denominators were reported for each analysis. Data were recorded as categorical or summary variables and analyzed descriptively to characterize the molecular type distribution within the study population.

### Preparation and preservation of isolates

The isolates were stored in Sabouraud dextrose agar supplemented with chloramphenicol in the fungal collection of the Laboratory of Mycology, INPA, and maintained in triplicate. In addition, isolates were preserved under mineral oil for future studies. Before molecular typing and phenotypic assays, each isolate was subcultured from the preserved stock, checked for purity by colony morphology and microscopy, and confirmed as viable under standardized conditions to obtain young and pure colonies for DNA extraction and inoculum preparation. Reactivation was performed on Sabouraud dextrose agar or YPD agar at 30 °C for 24–48 h, and cultures were checked for purity before DNA extraction and phenotypic assays.

### DNA extraction

DNA extraction was performed to obtain genomic DNA for URA5 PCR-RFLP. DNA was extracted from reactivated fungal cultures using the phenol-chloroform method described by Ferrer et al. [20], with minor modifications restricted to biomass preparation and incubation steps. DNA concentration and purity were determined by UV spectrophotometry at 260/280 nm using a BioSpectrophotometer (Eppendorf, Hamburg, Germany). DNA quality was assessed by spectrophotometry and by successful amplification of the URA5 target. Samples with poor amplification or doubtful purity were re-extracted before molecular typing.

### Molecular typing: which *Cryptococcus* spp. molecular types circulated in Amazonas?

Genotyping was performed using URA5 as the molecular target according to the PCR-RFLP method described by Meyer et al. [9]. PCR reactions included DNA template, buffer, MgCl2, dNTPs, URA5-F and URA5-R primers, and Taq DNA polymerase (Invitrogen/Thermo Fisher Scientific, Waltham, MA, USA). Each PCR run included molecular-type reference strains and a no-template negative control. RFLP analysis was performed with Sau96I and HhaI enzymes, and molecular-type assignment was based only on reproducible URA5 PCR-RFLP profiles visually matched with reference strains representing VNI, VNII, VNIII, VNIV, VGI, VGII, VGIII, and VGIV.

### Phenotypic comparison according to molecular type

Because phenotypic assays required viable isolates, standardized inoculum preparation, a stratified 30-isolate subset comprising 24 VNI and all 6 VGII isolates was evaluated for phospholipase activity and melanization. Fluconazole MIC data were analyzed separately for the complete 70-isolate dataset. Genotype-phenotype comparisons were considered descriptive and hypothesis-generating, because the VGII subgroup was small.

### Fluconazole MIC assessment

Fluconazole MICs were determined for all 70 isolates by broth microdilution according to CLSI M27-A4 [21]. The MIC dataset of the isolates was retrieved from the laboratory susceptibility database established for the work by Moreira et al. [22] and subsequently expanded using the same standardized protocol. Individual MIC values, final molecular types, specimen source, and method-specific metadata are provided in the Supplementary Table S1. Because the VGII subgroup comprised only six isolates, MIC comparisons between molecular types were considered descriptive, and formal genotype-associated inference analysis was not conducted.

### Evaluation of extracellular phospholipase activity

Phospholipase activity was evaluated on egg-yolk agar using a plate-based method originally described for pathogenic yeasts [23] and interpreted as a comparative extracellular phospholipase assay adapted to *Cryptococcus* spp. under the tested conditions. Its biological relevance in *C. neoformans* pathogenesis has been supported by experimental studies [24]. Spot inoculation was performed in triplicate, plates were incubated at 30 °C for up to 96 h, and activity was expressed using the Pz index.

### Evaluation of melanin production

Melanin production was evaluated using sunflower seed agar [25], and its interpretation as a virulence-associated phenotype was based on the recognized contribution of melanin to microbial pathogenesis [19]. Previously reactivated isolates were standardized to approximately 1 × 10^6 cells/mL, inoculated onto the medium, and incubated at 30 °C. Melanization was evaluated semiquantitatively using an ordinal scale: 0, absence of pigmentation; 1+, slight pigmentation; 2+, moderate pigmentation; 3+, intense pigmentation; and 4+, very intense dark brown to black pigmentation. Readings were performed after 24 h and 96 h. Melanization on sunflower seed agar was interpreted as a semiquantitative screening phenotype and not as a direct measure of clinical virulence.

### Pz index and statistical analysis

For enzyme assays, the Pz index was calculated as the colony diameter divided by the total diameter, including the colony plus the surrounding enzymatic precipitation or degradation zone [23, 24]. Lower Pz values indicated stronger enzymatic activity. Measurements were performed in millimeters using the same reading time for all isolates within each assay, and mean values of the technical replicates were used for the analyses. Percentages were reported with one decimal place for the larger VNI group, whereas categorical VGII results were preferentially presented as absolute proportions because this subgroup included only six patients. Pz values were reported with two or three decimal places. Because MIC values were measured on a twofold dilution scale, they were summarized using geometric means and observed minimum-maximum ranges. No inferential statistical comparisons were performed, because the VGII subgroup was small and the phenotypic analyses were considered as exploratory.

## Results

### Molecular types of clinical *Cryptococcus* spp. isolates from Amazonas, 2020–2024

To address the first objective, URA5 PCR-RFLP identified only two molecular types among the 70 isolates: VNI predominated, accounting for 64/70 isolates (91.4%), whereas VGII was detected in 6/70 isolates (8.6%). No VNII, VNIII, VNIV, VGI, VGIII, or VGIV profiles were detected in this clinical collection.

**Fig. 1:**
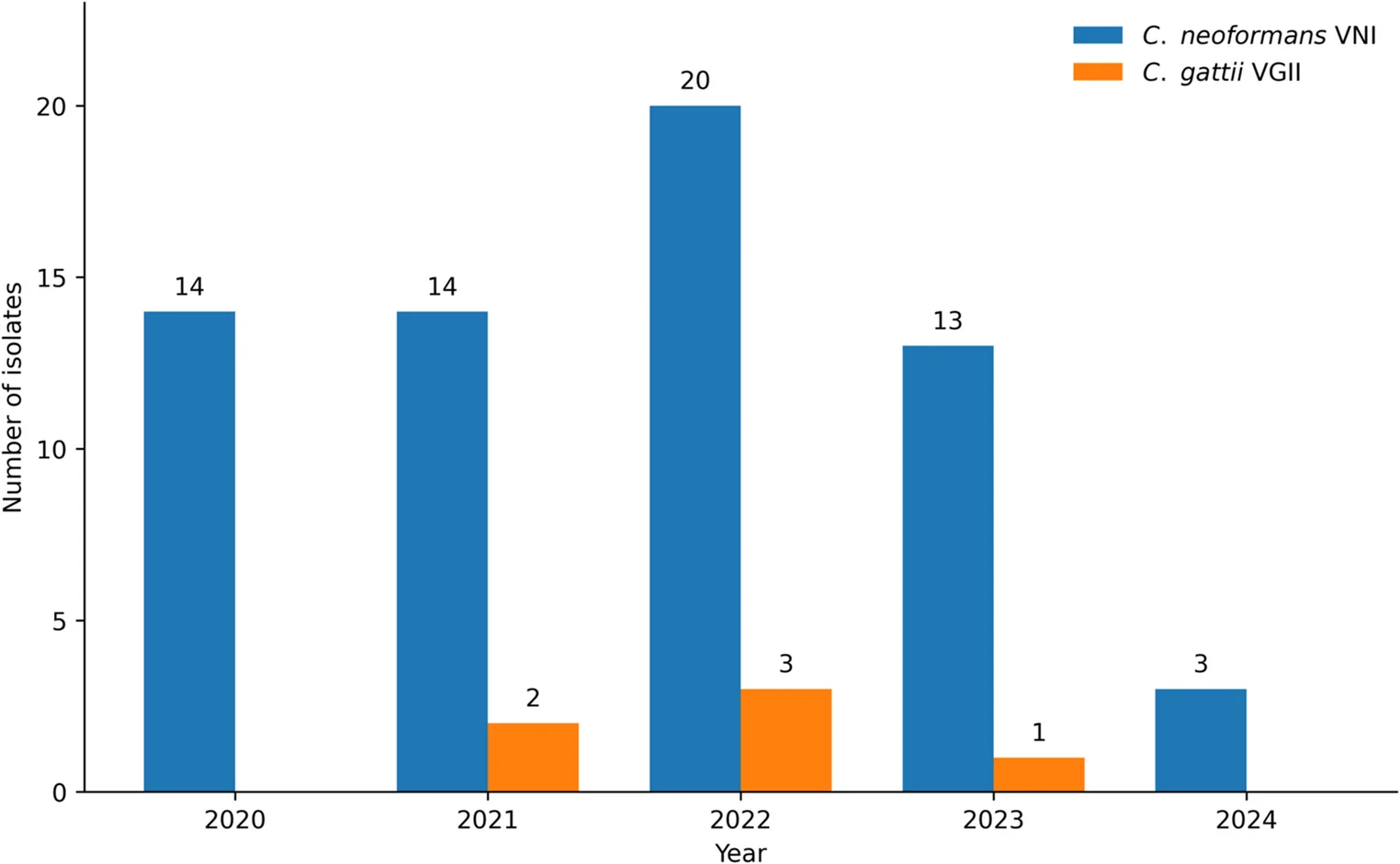
Annual distribution of *Cryptococcus* spp. molecular types identified among clinical isolates from Amazonas, Brazil, 2020–2024. The molecular type VNI predominated throughout the study period (64/70 isolates), whereas VGII was identified in 2021, 2022, and 2023 (6/70 isolates)

To evaluate temporal distribution, molecular types were grouped by year of isolation. VNI was detected throughout the study period, with 14 isolates in 2020, 14 in 2021, 20 in 2022, 13 in 2023, and 3 in 2024. VGII was detected in 2021, 2022, and 2023 (Fig. 1). The lower number of isolates in 2024 should be interpreted cautiously because isolate availability and data completeness differed from previous years.

### Clinical characteristics of patients according to the two main molecular types

The case series was predominantly composed of male, young adult, and HIV-positive patients. Men accounted for 49/70 cases, HIV infection was recorded in 65/70 patients, and in-hospital death occurred in 17/70 patients (Table 1). Detailed demographic and clinical distributions stratified by molecular type are presented in Table 1.

**Table 1:** Demographic and clinical characteristics of patients with cryptococcosis according to molecular type, Amazonas, Brazil, 2020–2024

Variable	Category/measure	VNI (*n* = 64)	VGII (*n* = 6)
Sex	Female, n (%)	18 (28.1)	3/6
	Male, n (%)	46 (71.9)	3/6
Age in years	Median (IQR)	31.5 (26.8–41.3)	35.0 (20.8–43.3)
Age group	≤ 20 years, n (%)	2 (3.1)	2/6
	21–30 years, n (%)	27 (42.2)	0/6
	31–40 years, n (%)	17 (26.6)	2/6
	41–50 years, n (%)	11 (17.2)	1/6
	51–60 years, n (%)	7 (10.9)	1/6
	> 60 years, n (%)	0 (0.0)	0/6
HIV infection	Yes, n (%)	63 (98.4)	2/6
	No, n (%)	1 (1.6)	4/6
Initial CD4 count, cells/µL	Median (IQR); available n	25 (14–56); *n* = 61	43.5; *n* = 2*
Initial CD4 count < 100 cells/µL	n/N (%)	51/61 (83.6)	2/2
ICU admission	Yes, n (%)	16 (25.0)	2/6
	No, n (%)	48 (75.0)	4/6
Hospital outcome	Hospital discharge, n (%)	48 (75.0)	5/6
	In-hospital death, n (%)	16 (25.0)	1/6
Recurrence	Yes, n/N (%)	15/63 (23.8)	1/6
	No, n/N (%)	48/63 (76.2)	5/6

Percentages are reported for the VNI group; categorical VGII results are presented as absolute proportions because this subgroup included only six patients. CD4 denominators are shown, because baseline CD4 + T-lymphocyte counts were not available for all patients. The interquartile range was not reported for CD4 + T-lymphocyte counts of the VGII group, because only two baseline values were available. Recurrence information was available for 63/64 VNI patients and all six VGII patients. No inferential comparison was performed, because the VGII group was small. *Only two baseline CD4 values were available in the VGII subgroup; therefore, the median is presented without an IQR

*IQR*, interquartile range; *ICU*, intensive care unit; *VNI*, *Cryptococcus neoformans* molecular type VNI; *VGII*, *Cryptococcus gattii* molecular type VGII

When stratified by molecular type, 63/64 VNI patients were HIV-positive, whereas 2/6 VGII patients were HIV-positive and 4/6 were HIV-negative. In-hospital death occurred in 16/64 VNI cases and 1/6 VGII cases. Because the VGII group included only six cases, these findings were considered as descriptive and should not be interpreted as evidence of genotype-associated risk

### Fluconazole MIC records and exploratory virulence-associated phenotypes according to molecular type

**Fig. 2:**
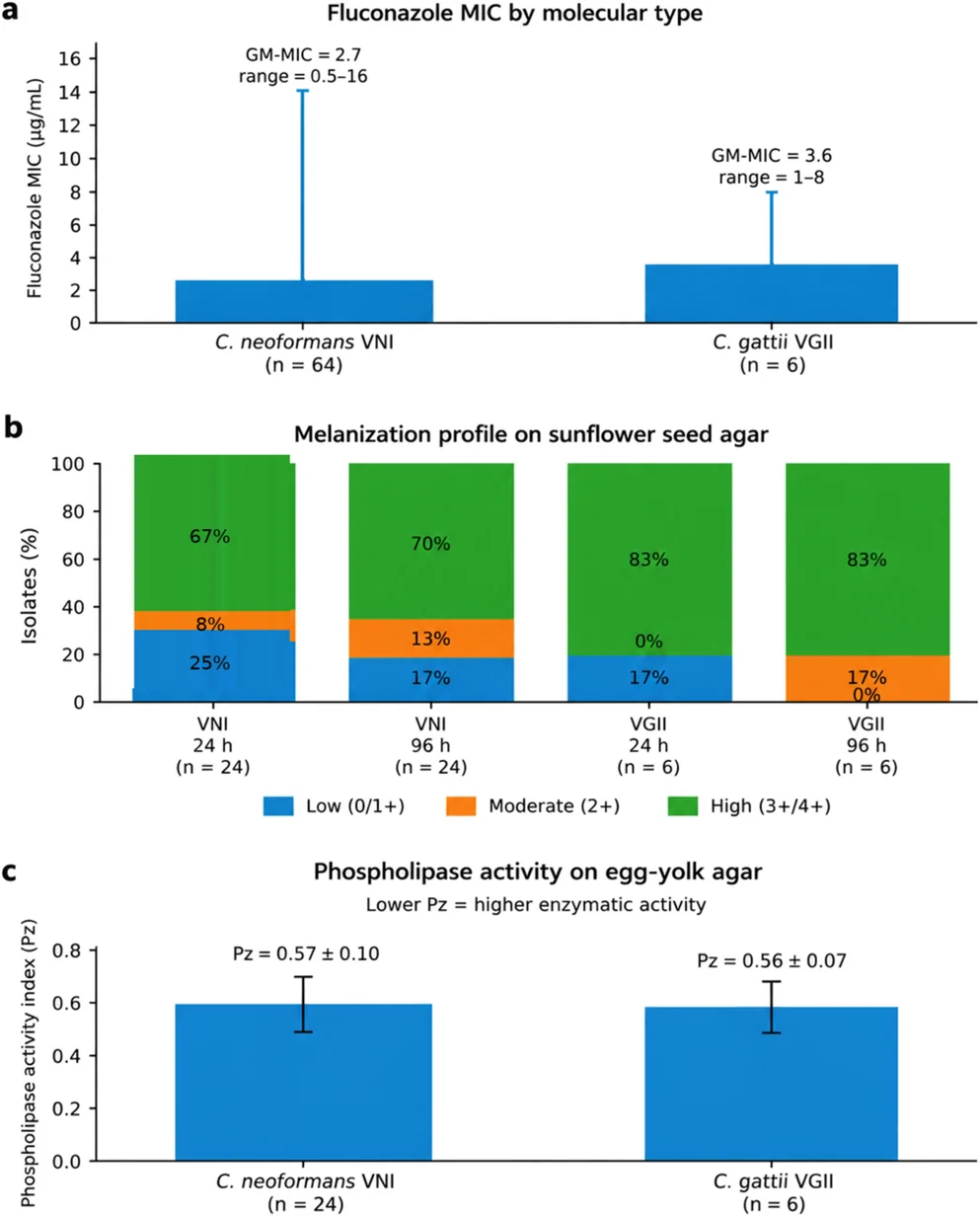
Fluconazole susceptibility and exploratory virulence-associated phenotypes of clinical *Cryptococcus* spp. isolates according to molecular type. (**a**) Fluconazole MICs of 64 VNI and 6 VGII isolates, determined by broth microdilution according to CLSI M27-A4 and presented as geometric means with observed minimum-maximum ranges. Individual MIC values, specimen source, molecular types, and method metadata are provided in Supplementary Table S1. (**b**) Melanization profiles on sunflower seed agar at 24 and 96 h were evaluated in a 30-isolate subset. (**c**) Phospholipase activity on egg-yolk agar, expressed using the Pz index (lower Pz values indicate higher enzymatic activity), was also evaluated in this 30-isolate subset. Owing to the small number of VGII isolates, comparisons between molecular types were assessed descriptively only

To explore phenotypic differences between VNI and VGII isolates, fluconazole MICs and selected virulence-associated phenotypes were summarized descriptively. Fluconazole MICs were analyzed for the complete 70-isolate dataset as a susceptibility-related parameter. Phospholipase activity and melanization were evaluated as exploratory virulence-associated phenotypes in the stratified 30-isolate subset comprising 24 VNI and 6 VGII isolates. The comparative analysis of the results is presented for fluconazole MICs in panel a, melanization in panel b, and phospholipase activity in panel c (Fig. 2).

Fluconazole MICs as determined applying broth microdilution according to CLSI M27-A4 were available for all 70 isolates. VNI isolates (*n* = 64) showed a geometric mean MIC of 2.7 µg/mL (observed range, 0.5–16 µg/mL), whereas VGII isolates (*n* = 6) presented a geometric mean MIC of 3.6 µg/mL (observed range, 1–8 µg/mL) (Fig. 2a). Individual isolate-specific values are provided in the Supplementary Table S1. Although the geometric mean was numerically higher among VGII isolates, this difference should be interpreted cautiously, because the VGII subgroup included only six isolates. Accordingly, MIC values were analyzed descriptively, without formal genotype-associated inference analysis. Melanization was phenotypically evaluated in the 30-isolate subset. It was frequently observed in case of both molecular types. The complete categorical distribution of low (0/1+), moderate (2+), and high (3+/4+) melanization at 24 and 96 h is shown in Fig. 2b. Differences between molecular types were interpreted descriptively and not as evidence of a robust genotype-associated effect.

## Discussion

The study was conducted to address three questions on the epidemiology of *Cryptococcus* spp. in the Brazilian Amazonas region: molecular-type distribution, patient profile according to molecular type, and exploratory phenotypic differences between VNI and VGII. The main robust finding was the predominance of *C. neoformans* VNI among culture-confirmed clinical *Cryptococcus* spp. isolates from Amazonas, Brazil. VNI accounted for 64/70 isolates (91.4%), whereas *C. gattii* VGII was detected in 6/70 isolates (8.6%). Therefore, the study provides a regional culture-based baseline for cryptococcosis surveillance in patients from the Brazilian Amazon.

First, the predominance of *C. neoformans* VNI was the clearest microbiological result. VNI was detected across the study period, confirming that this molecular type is the dominant cause of culture-confirmed cryptococcosis in this Amazonas series. This finding agrees with global, Latin American, and Brazilian reports showing the predominance of VNI in HIV-associated cryptococcosis, while the detection of VGII confirms that the *C. gattii* species complex also circulates among clinical cases in the region [6–11, 17, 18].

Second, the clinical profile explains much of the molecular distribution observed in this study. Most patients were young adult men living with HIV, and CD4 counts were very low among those with available data, indicating advanced immunosuppression. VNI was strongly associated with HIV-positive cases, whereas VGII included proportionally more HIV-negative patients. However, the small number of VGII cases prevents any conclusion about genotype-associated severity. The 24.3% in-hospital mortality should therefore be interpreted mainly as a marker of the severity of cryptococcosis in this reference-center population, not as an effect of molecular type [26–29].

Third, the exploratory phenotypic assays did not demonstrate a robust difference between VNI and VGII isolates. Fluconazole MICs were determined using a standardized broth microdilution method and were similar in magnitude in comparison of the molecular types. Phospholipase activity showed closely overlapping Pz values. Melanization was frequent in both groups, with a seemingly higher proportion of isolates showing high melanization levels among VGII isolates. However, the small VGII subgroup discourages statistical or biological overinterpretation. Insofar, these phenotypic data should be considered as hypothesis-forming, but they did not provide evidence on clinical virulence or predict patient outcomes in this retrospective assessment [12–19, 22, 25–31].

The joint assessment of molecular typing and phenotypic data is particularly important in regions where cryptococcosis remains under-characterized. In Amazonas, fungal isolate preservation linked to clinical information can support retrospective analyses, identify temporal patterns, and generate hypotheses about host-pathogen interactions. The presence of both VNI and VGII in the clinical collection indicates that regional surveillance should not be limited to a single species complex. Future work should integrate culture, cryptococcal antigen testing, direct microscopy, specimen source, standardized antifungal susceptibility testing, MLST or whole-genome sequencing, environmental sampling, and broader phenotypic characterization, including thermotolerance at 37 °C and capsule-size measurement, to clarify transmission dynamics and the relationship between molecular type, phenotype, and clinical outcome [7–11].

Although this study was not designed to identify predictors of fatal courses, the recorded 24.3% in-hospital mortality highlights the clinical severity of cryptococcosis in this setting. Previous cohorts have shown that mortality is influenced by clinical and microbiological markers that were not available for all patients in the present dataset, including fungal burden, cerebrospinal fluid opening pressure, mental status, bloodstream dissemination, immune status, timing of antifungal therapy, and early fungicidal activity [26–29]. Treatment-related information was abundant in some records but generally incomplete, heterogeneous, and insufficiently standardized for comparative analysis. This particularly applied to time to antifungal initiation, induction regimen, drug dosage, and management of intracranial pressure. Therefore, outcome interpretation must remain descriptive in this analysis.

The study has important limitations. It was retrospective; clinical variables depended on medical-record completeness; the VGII group was small; phenotypic assays were performed in a subset of isolates; and URA5 PCR-RFLP does not replace MLST or whole-genome sequencing, which could not be conducted due to funding constraints. The small number of VGII isolates limited statistically meaningful comparisons of fluconazole MIC distributions and virulence-associated phenotypes between the identified molecular types. Treatment-related information was incomplete, heterogeneous, and insufficiently standardized for comparative analyses. Thermotolerance at 37 °C and capsule-size measurements, both classic *Cryptococcus* spp. virulence-associated phenotypes, were not included in the available experimental dataset and should be considered for future prospective studies. The latter should integrate standardized clinical variables, information on antifungal therapy, cryptococcal antigen titers, fungal burden, opening pressure, molecular typing, standardized susceptibility testing, environmental sampling, and systematically documented patient outcomes.

Nevertheless, the findings provide a practical regional baseline: VNI predominates among clinical isolates from Amazonas, VGII circulates at lower frequency, and retrospective phenotypic assays should guide future prospective studies rather than define clinical risk. The robust result is molecular epidemiological, whereas virulence-associated phenotypes should be interpreted as exploratory.

## Conclusion

This study established a clinical isolate-based molecular baseline for cryptococcosis surveillance in Amazonas, Brazil. The molecular type VNI was the predominant molecular type among 70 clinical isolates, whereas VGII was detected less frequently. The corresponding patients were predominantly HIV-positive young adults, and in-hospital mortality was high. Standardized fluconazole MICs, phospholipase activity, and melanization did not show robust phenotypic differences between the molecular types VNI and VGII. Prospective surveillance integrating molecular typing, standardized susceptibility testing, key prognostic variables, and patient outcomes is recommended.

## Supplementary Information

Below is the link to the electronic supplementary material.

Supplementary File 1 (XLSX 10.9 KB)

## Data Availability

De-identified isolate-level data supporting the fluconazole MIC analysis, including study isolate identifiers, molecular types, specimen source, individual MIC values, and testing method, are provided in Supplementary Table S1. Additional clinical data are available from the corresponding author upon reasonable request, subject to ethics approval, institutional regulations, and patient-confidentiality safeguards.

## Declarations

### Use of AI-assisted tools

AI-assisted tools were used only for language editing, readability, and formatting support. All scientific content, analyses, interpretation, and final decisions were produced, reviewed, and approved by the authors, who take full responsibility for the integrity and accuracy of the manuscript.

### Materials availability

Clinical isolates are maintained in the fungal collection of the Laboratory of Mycology, National Institute for Amazonian Research (INPA), and may be made available for collaborative research upon reasonable request and subject to institutional, ethical, and biosafety requirements.
